# Regulatory Roles of Dclk1 in Epithelial Mesenchymal Transition and Cancer Stem Cells

**DOI:** 10.4172/2157-2518.1000257

**Published:** 2016-03-07

**Authors:** P Chandrakesan, J Panneerselvam, D Qu, N Weygant, R May, MS Bronze, CW Houchen

**Affiliations:** 1 Department of Medicine, University of Oklahoma Health Sciences Center, Oklahoma City, OK 73104, USA; 2 Stephenson Oklahoma Cancer Center, University of Oklahoma Health Sciences Center, Oklahoma City, OK 73104, USA; 3 Department of Veterans Affairs Medical Center, Oklahoma City, OK 73104, USA; 4 Department of Pathology, University of Oklahoma Health Sciences Center, Oklahoma City, OK 73104, USA; 5 COARE Biotechnology, Oklahoma City, OK, USA

**Keywords:** Cancer stem cells, Malignancy, Carcinogenesis

## Abstract

The identification of functionally relevant subpopulations of therapy-resistant cancer cells is a challenge. These cells, intrinsically resistant to conventional therapy, can cause recurrence. Evidence has suggested that therapy-resistant cancer cells are likely epithelial–mesenchymal transition (EMT) cells and/or stem-like cells called cancer stem cells (CSCs). EMT, a normal embryological process that converts epithelial cells into mesenchymal cells, is frequently activated during cancer development and progression. CSCs are a small subpopulation of cancer cells within a tumor mass that have the ability to self-renew and maintain tumor-initiating capacity by giving rise to heterogeneous lineages of cancer cells that comprise the whole tumor. Although the origin of CSCs and EMT cells remains to be fully explored, a growing body of evidence has indicated that the biology of EMT and CSCs is strongly linked. Doublecortin-like kinase 1 (DCLK1), a cancer stem cell marker, is functionally involved in maintaining cancer stemness and the process of EMT important for cancer initiation, cancer metastasis, and secondary tumor formation. Therefore, targeting these cells may provide new strategies to overcome tumor heterogeneity, therapeutic resistance, and cancer relapse. In this review, we will provide a potential mechanistic link between EMT induction and the emergence of CSCs for the origin and progression of cancer. We will highlight the functional activity of DCLK1 in supporting EMT and cancer cell self-renewal, which will lead us to a better understanding of DCLK1 expression in cancer development and progression, and help us to develop targeted therapies for effective cancer treatment.

## Introduction

**Figure 1:**
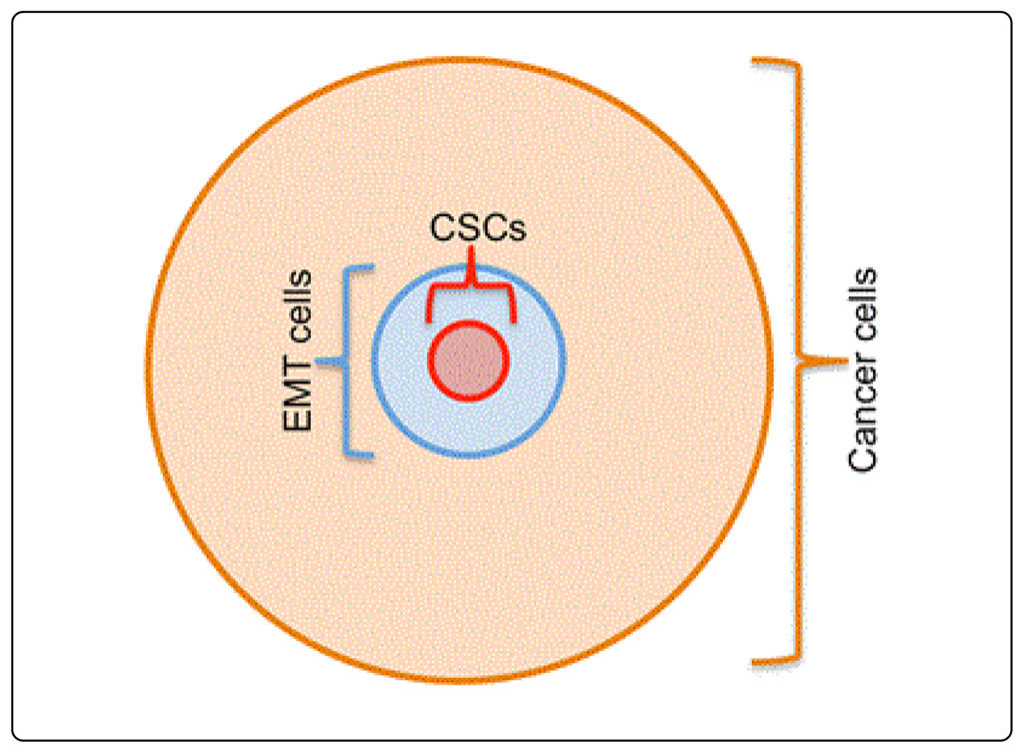
Existence of EMT-type cells and CSCs in cancer tissues.

Although cancer mortality has steadily declined over the past decade, primarily due to earlier detection and adjuvant and targeted therapies, tumor recurrence remains a major cause of morbidity and mortality [1,2]. Therefore, novel therapies that prevent treatment resistance, relapse, and metastasis are required. Tumors are widely accepted to contain a subpopulation of cells called CSCs that have the ability to self-renew and regenerate the tumor [3,4]. The tumor and its microenvironment also contain unique EMT cells, which can survive in the peripheral circulation and actively cause relapse [5]. Populations of CSCs and EMT-type cells are small in cancer tissue; there are generally more EMT-type cells than CSCs (Figure 1). Recent evidence demonstrates that these cells have an intrinsic resistance to radio- and/or chemotherapy [2]. Both cell types link together in their phenotype and functions to facilitate cancer growth, metastasis, and recurrence [2,6].

CSCs are a small sub-population of cancer cells. EMT-type cells are a distinct small population of cancer cells within and around cancer tissue. There are more EMT-type cells than CSCs. CSCs are highly resistant to therapies, are long-lived, self-renew, and are involved in recurrence. EMT-type cells display moderate to maximum resistance to treatments and can self-renew. Many functional properties of EMT cells and CSCs overlap during cancer progression and metastasis.

Residual tumors after standard therapies are enriched for CSCs and have gene signatures with hallmarks of EMT-like properties [5]. Chemo- or radio-resistant tumors are reported to have high numbers of EMT-transformed CSCs [3,7]. Taken together, these findings suggest that EMT links CSCs, helping these cells to survive even in the peripheral circulation and actively causing relapse. This review will focus on the link between EMT and CSCs, since a better understanding of these links that are critically involved in cancer resistance, metastasis, and recurrence could lead the way to the development of effective therapies. Furthermore, this review will discuss the signaling mechanism linking EMT and CSCs, and discuss how the critical regulator Doublecortin-like kinase 1 (DCLK1) supports the EMT process and stemness for cancer development and progression.

DCLK1 is a member of the protein kinase super family and the doublecortin family, and marks colon and pancreatic cancer stem cells [8–10]. DCLK1 is overexpressed in many cancers, including colon, pancreas, liver, kidney [11], and esophageal cancer [9,12–15]. Studies from us and others supported that DCLK1 expression is critical for cancer growth, EMT, metastasis, and cancer cell self-renewal [9,14,16–18]. The functional interdependence between EMT-associated transcription factors and enhanced self-renewal highlights the common mechanism involved in their regulation. Therefore, highlighting the regulatory role of DCLK1 supporting EMT and CSCs will enhance our understanding of drug targets and help us to design novel and effective targeted therapies.

## Epithelial Mesenchymal Transition (EMT) and Cancer

EMT is a fundamental developmental process during which polarized epithelial cells convert into motile mesenchymal-like cells. This reversible process enables cells to move into the interior of the embryo, travel long distances, and participate in the formation of internal organs [19]. Although the EMT program is necessary for normal development and injury repair, the abnormal activation of EMT contributes to various pathologic conditions, including fibrosis and cancer initiation and progression [20,21]. The molecular signaling factors associated with EMT indicate that EMT is indispensible for tumor cells to bypass apoptosis, anoikis, and cellular senescence, and to escape immune surveillance for survival and to gain motility for metastasis [20,22].

Understanding and targeting the adaptive growth of EMT-driven cancer cells and/or EMT factors in cancer cells could extend progression-free survival of patients with cancer [22]. The activation of this transdifferentiation program depends on a network of EMT-inducing transcription factors that regulate the expression of proteins involved in loss of apical–basolateral polarity, cell–cell junction, cytoskeleton structure, and extracellular matrix degradation, including the repression of key epithelial genes, resulting in the formation of migratory cells with invasive properties [23,24]. The canonical EMT program is characterized by complex gene expression changes. A dominant consequence of these gene expression events is the upregulation of transcriptional repressors, such as the C2H2-type zinc-finger proteins Snail, Slug, and Zeb1, Zeb2/SIP1, and the bHLH factors Twist and E47 [25–27]. These transcriptional factors bind to E-box elements in the promoter of the gene encoding the adherens junction-protein E-cadherin, where they recruit histone deacetylases (HDACs) and other corepressors to assist chromatin condensation and subsequent transcriptional repression of E-cadherin expression [28].

The loss of E-cadherin expression is considered a key event in EMT. Loss of E-cadherin expression causes adherens junction breakdown. Loss of cell polarity follows. A significant concern regarding E-cadherin alteration is the counteracting role of another classical cadherin, the neural-type or N-cadherin [28]. Aberrant de novo expression of N-cadherin has been noted in many carcinomas from the breast, pancreas, colon, prostate, bladder, and head and neck regions, where N-cadherin complements a downregulation of E-cadherin [28]. Thus, the EMT cells can be distinguished by analyzing the expression of classical markers that are used to define either epithelial or mesenchymal characteristics. For example, epithelial cell markers include E-cadherin and ZO-1, whereas mesenchymal markers include fibronectin and vimentin [29,30].

**Figure 2:**
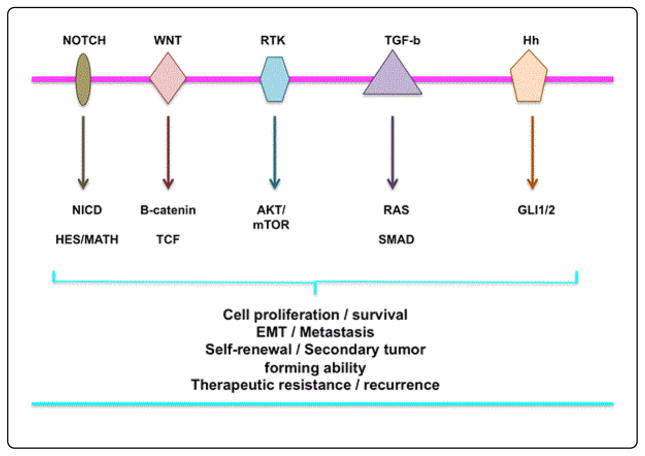
Overview of molecular signaling pathways governing EMT and CSCs.

Like EMT properties, the loss of polarity and gain of motility have also been ascribed to normal stem cells (NSCs) and CSCs [2]. Recent studies have found that multiple signaling pathways are involved in processing EMT in tumor cells. The phosphatidylinositol 3 kinase (PI3K)/AKT signaling pathway has emerged as a vital component of EMT. Furthermore, the Wnt/β-catenin, Notch, and NfKB signaling pathways also play important roles in the process of EMT in tumor cells [23]. However, these signaling pathways are also involved in regulating the self-renewal of CSCs and/or cancer stem-like cells for cancer survival and recurrence (Figure 2). Transforming growth factor β (TGF-β)/SMAD1 is also an important signaling pathway in EMT [31]. However, how the process of EMT and gain of stemness link with CSC is not well understood.

Overview of signaling pathways regulating cancer stemness, survival, proliferation, differentiation, EMT, resistance, and recurrence. The NOTCH, WNT, RTK, TGF-B, and Hedgehog signaling pathways have been implicated in the development and maintenance of EMT-type cells and CSCs. These signaling pathways and their downstream targets function as the novel regulatory mechanisms that may promote self-renewal, EMT, and differentiation, and thereby provide avenues for therapeutic interventions. These signaling pathways are uncontrolled in cancer cells, but are likely coordinated for cancer cell growth. AKT, protein kinase B; Hh, Hedgehog protein; mTOR, mammalian target of rapamycin; NICD, RAS, RTK, receptor tyrosine kinase; TCF, T cell factor transcription factor; TGF-B, Transforming growth factor beta; Wnt, Wingless.

## Cancer Stem Cells (CSCs)

Stem cells reside at the top of the cellular hierarchy, and are involved in the maintenance and repair of many adult tissues. Stem cells have two principal properties: the ability to self-renew and differentiate into dedicated cell types, and the ability to home towards sites of pathology and malignant lesions [32,33]. CSCs are defined as a subpopulation of cancer cells that bear properties of stem cells and constitute a pool of self-sustaining cells with the exclusive ability to cause the heterogeneous lineages of cancer cells; CSCs show the greatest diversity in cancer progression [34,35]. The presence of CSCs was first established in acute myelogenous leukemia, and later demonstrated in breast, pancreatic, and brain tumors [36–39]. Although CSCs share functions with normal stem cells, there are unanswered questions, such as whether CSCs arise from normal stem cells, from progenitor cells, or due to genetic alterations in differentiated cells.

However, CSCs are also hypothesized to arise from normal stem cells and from differentiated cells. This hypothesis has not been tested. Therefore, identifying the molecular characteristics of CSCs is urgently needed to understand their origin, and to allow for the development of effective therapeutics targeting CSCs. Isolating CSCs can provide enormous information on their molecular structure and characteristics. However, the isolation of CSCs is a challenge due to lack of definite cell surface markers. The identification of CSCs in various malignancies has also revealed that CSCs are largely tissue-specific and that a universal CSC marker is unlikely [40].

Recent studies have proposed several methods and fundamental concepts regarding CSCs markers. CD44+CD24−/low, CD44+CD24−/lowESA+, CD133+, and ALDH1+ cancer cells are CSC candidates with high tumor-initiating and cancer formation abilities *in vitro* and *in vivo* [40,41]. However, whether CD44+CD24−/low, CD44+CD24−/lowESA+, CD133+, and ALDH1+ cancer cells represent distinct CSC populations, and whether they represent the origin of these cells, remain unknown.

Fluorescence-activated cell sorting (FACS) and human tumor xenograft models in immune-deficient mice play important roles in the evaluation of the characteristics of isolated CSCs. Anchorage independent growth (clonogenic assay) is also considered an effective way to isolate and delineate CSCs characteristics [9,42]. These procedures are recognized as valid gold standard approaches to identify CSCs in cancer. In addition to the functional assays of xenograft models *in vivo* and clonogenic assays *in vitro* to analyze the characteristics of CSCs, the evaluation of gene and/or protein expression of “stemness” genes and/or pluripotency factors will be of great utility in identifying CSCs.

Signaling pathways identified in normal stem cells pave the way for the elucidation of CSC signaling systems. The Notch, Wnt, PTEN, hedgehog, NFkB, and (PI3K)/Akt signaling pathways have already been confirmed to play critical roles in CSCs [43–45]. These signaling pathways have also been associated with the regulation of diverse cellular functions of cancer, including growth, survival, metastasis, angiogenesis, and tumorigenesis (Figure 2). Furthermore, these signaling pathways are reported to play critical roles in the process of EMT, demonstrating the link between EMT and CSCs [46,47].

## Links: EMT and CSCs

**Figure 3:**
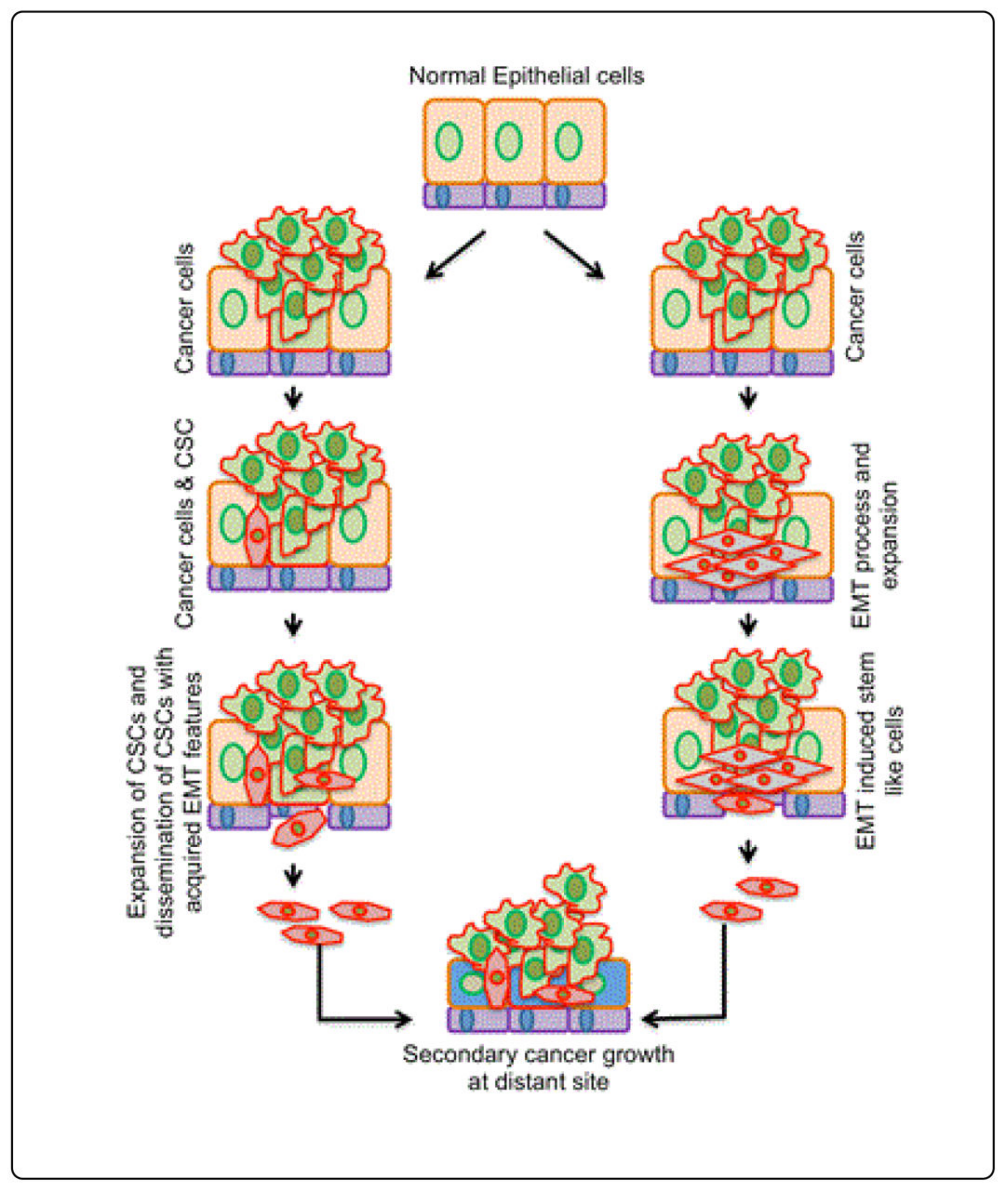
Epithelial-mesenchymal transition and stem cell traits in cancer progression.

The EMT process was shown to provide normal and transformed mammary epithelial cells with stem cell properties, including the ability to self-renew and to efficiently initiate tumors [48]. Furthermore, evidence connects the EMT process with the origin of CSCs and suggests EMT as a precondition for cancer metastasis [42,49,50]. CSCs frequently exhibit EMT properties in their dissemination to different sites for metastasis and secondary tumor development [6,51]. This shared link between EMT and CSCs might have significance in tumor initiation, progression, and recurrence (Figure 3) [52,53].

Cancer originates from either normal adult tissue stem cells or from more differentiated progenitors that have acquired self-renewal capabilities. These stem cells or stem-like cells acquire EMT features to metastasize. Indeed, the EMT process likely occurs at differentiated cancer cells for migratory and invasive potential could also acquire self-renewal abilities. EMT cells can also display stem-like cell features and generate secondary cancers at distant sites. This model shows regulated co-ordination and/or a link between EMT and CSCs that generates secondary cancer at distant sites and promotes recurrence.

Stem cells isolated from normal mouse mammary tissues, human reduction mammoplasty tissues, and immortalized human mammary epithelial cells (HMLECs) were recently found to express markers associated with EMT [48]. When overexpressed with either of the transcription factors Snail or Twist, or exposed to cytokines, HMLECs generate malignancy with stem cell properties [54,55]. Researchers observed the acquisition of EMT properties, along with enrichment of cells expressing CSC markers (the CD44high/CD24low phenotype) and enrichment of stem cell function as assessed by an increased self-renewal ability to form increased mamospheres [55]. Induced expression of EMT factor Twist1 in mammary epithelial cells can generate EMT and stemness, thus linking EMT to cancer CSCs.

In an independent experiment, isolated CD44high/CD24low cells from neoplastic human breast tissues expressed higher EMT markers than the isolated CD44low/CD24high cells [48]. Thus, either the induction or generation of EMT or CSCs seems to produce stem-like cells with increased self-renewal and migratory abilities and initiate new tumors. Molecular signaling pathways, including Notch, Wnt, PTEN, hedgehog, NFkB, (PI3K)/Akt, and TGFβ, play vital roles in regulating EMT-associated transcriptional factors to suppress the expression of E-cadherin and induce EMT, resulting in tumor migration [23]. Moreover, these gene expression patterns also play critical roles in regulating stemness and self-renewal in cancer cells [56,57], suggesting that the de-differentiated cancer cells may link EMT properties with a stem-cell like phenotype to generate migrating CSCs as the basis of metastasis and to develop secondary cancers.

Stemness and EMT can also determined by a binary feedback loop in which two factors are mutually inhibited. LIN28/let-7 inhibition controls stemness [58]. However, miR-200/ZEB controls the EMT process [59]. These two segments are linked, because LIN28 is inhibited by miR-200 and both are triggered by Snail1, which inhibits let7 and activates ZEB [60,61]. Taken together, these findings are sufficient to suggest that EMT, which has a high probability of gaining stemness compared with the epithelial or mesenchymal state, and CSCs, which have a high probability of gaining EMT for metastasis, are linked for the development, progression, and metastasis of cancer. Therefore, it is necessary to better understand the factors regulating or supporting EMT and CSCs, which are the biggest challenges in cancer treatment.

## DCLK1: EMT and Cancer Stemness

Doublecortin-like kinase 1 (DCLK1) is a member of the protein kinase super family and the doublecortin family. DCLK1 is overexpressed in many cancers, including colon, pancreas, liver, esophageal, and kidney cancer [12,62–64]. Recent studies show that DCLK1 specifically marks tumor stem cells (TSCs) that self-renew and generate tumor progeny in ApcMin/+ mice [8,18]. Furthermore, ablation of DCLK1+ cells led to regression of intestinal polyps without affecting normal intestinal epithelial cell function [8].

These results coincide with our findings reporting that siRNA-based DCLK1 interference leads to growth arrest of colon and pancreatic cancers in xenograft models [13,14]. A recent study by Li and Bellows demonstrated increased DCLK1+ expression among cell fractions with a higher percentage of stem-like HCT116 human CRC cells [65]. Mirzaie et al. compared DCLK1 with Lgr5 and reported higher DCLK1 expression in blood samples from patients undergoing cancer treatment, suggesting that DCLK1 may be a more relevant CSC biomarker candidate [17].

**Figure 4:**
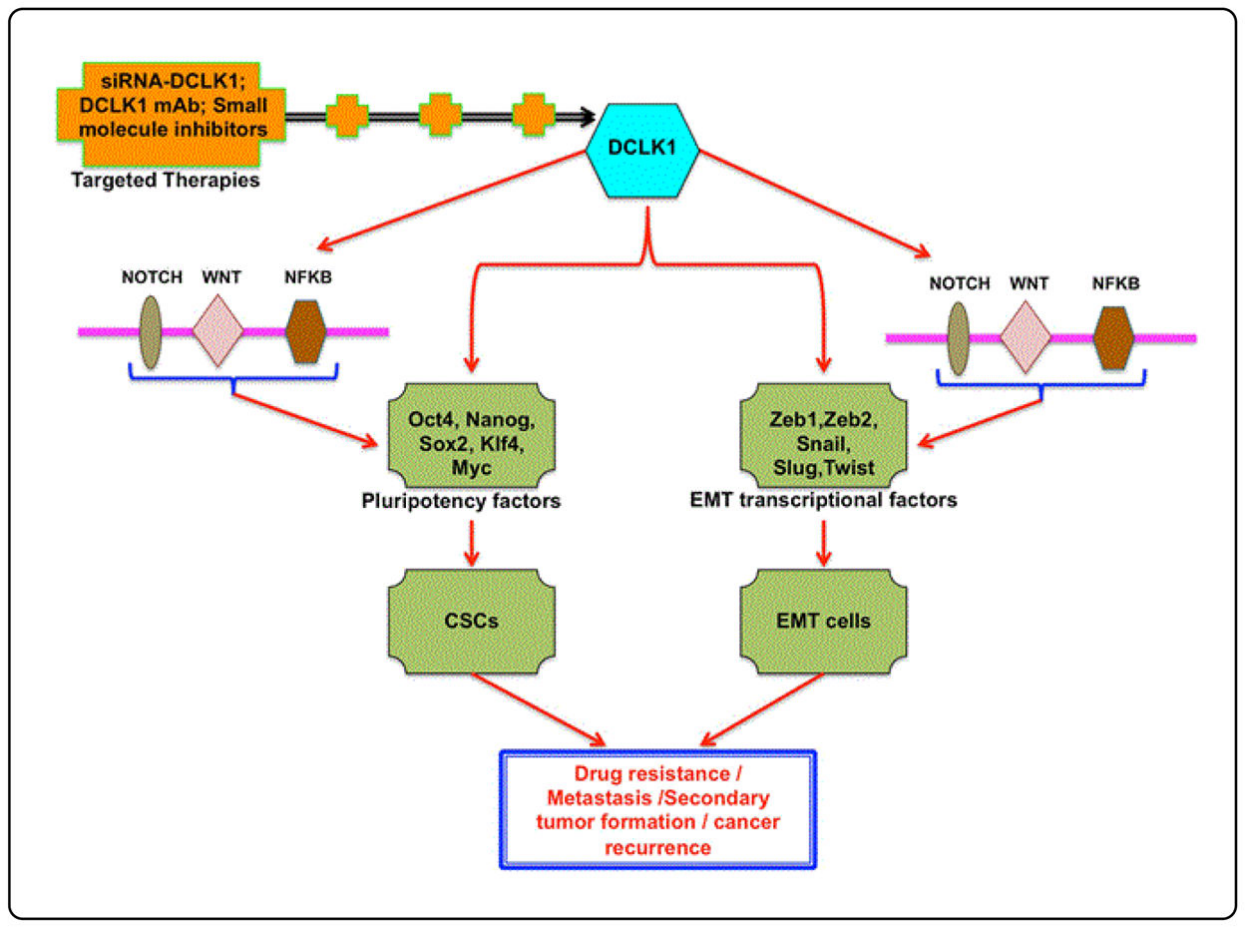
DCLK1 regulates EMT and CSCs: a novel target in cancer treatment.

More recently, we demonstrated that treatment with siDCLK1 incorporated into nanoparticles (siDCLK1-NP) significantly reduced the number and size of polyps, adenoma, and adenocarcinoma in ApcMin/+ mice model [9]. Furthermore, siDCLK1-NP treatment decreased the self-renewal and EMT of intestinal epithelial cells [9]. DCLK1 is now suggested to be a master regulator, regulating the pluripotency factors, including Nanog, Oct4, Sox2, Klf4, and Myc, that are critical for stemness of cancer cells and EMT transcriptional factors, including Snail, Slug, Twist, and Zeb1 for metastasis and survival in many solid tumors (Figure 4) [9,13–15,62,66,67]. Interestingly, the critical signaling pathways NOTCH, NFKB, and WNT that are involved in regulating both EMT and CSCs are controlled by DCLK1 expression in cancer models (Figure 4) [9,67]. Our collaborator reported that DCLK1+ cells are quiescent and are involved in cancer initiation upon injury [18]. The development and progression of pancreatic cancer have also been shown to depend on DCLK1+ TSCs [10]. Studies from us and others supported that DCLK1 expression is critical for cancer growth, EMT, and metastasis [9,10,12,14,15,18,62].

Taken together, these findings support the notion that DCLK1 is critical for cancer initiation, growth, stemness, EMT, and metastasis, and that DCLK1+ cells can identify cancer stem-like cells that are critical for therapy resistance and cancer recurrence (Figure 4). Therefore, targeting DCLK1 with novel therapeutic strategies is a promising approach. Small molecule DCLK1 inhibitors siDCLK1-NP and ADC have the potential to reduce cancer initiation, progression, and metastasis by regulating EMT and CSC.

DCLK1 is overexpressed in many solid tumors and cancers. DCLK1 regulates pluripotency factors and EMT-associated transcriptional factors, thus regulating CSCs and EMT cells. CSCs and EMT cells are involved in cancer development and progression, and are critical to drug resistance and relapse. DCLK1 also regulates the NOTCH, NFKB, and WNT molecular signaling pathways that promote cancer growth and progression and support EMT and CSCs. Conventional therapies target proliferating cancer cells and also kill normal cells. Therefore, we urgently need efficient targeted therapies. Researchers suggest that DCLK1 may suppress EMT, metastasis, and CSCs in early and even advanced stage cancer. DCLK1-targeted treatment may increase patient survival.

## Development of Novel Therapeutic Strategies

Accumulating evidence suggests that the conventional chemo- and radiotherapy for cancer largely targets differentiated tumor cells, which comprise most of the primary tumor [68,69]. Conventional treatments are not ideal, since they have partial tumor specificity, severe toxicity, and/or lead to the development of resistance. These therapies can lead to considerable tumor reduction, but are largely ineffective at preventing tumor recurrence [68,70]. The cells that exhibit intrinsic resistance to conventional radio- and/or chemotherapies have the characteristics of stem cells and are called CSCs.

**Figure 5:**
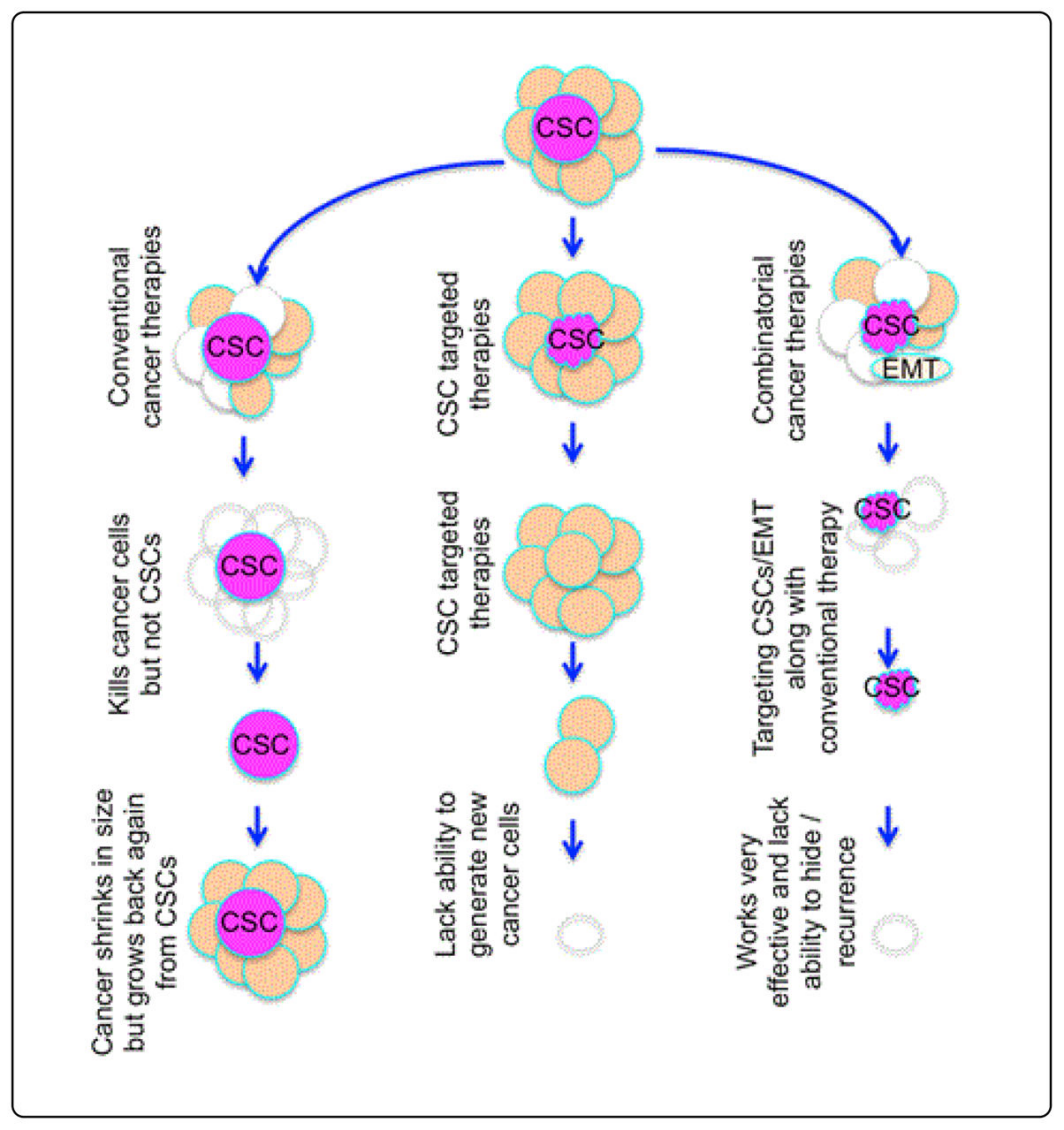
Novel strategies in cancer treatment.

Tumor regrowth is likely mediated by CSCs, EMT cells, or both. Both cell types have increased resistance to standard therapies and cell death. Increasing evidence indicates that EMT properties or CSCs are connected with aggressive cancer subtypes and poor clinical outcomes for patients with breast cancer [70–72]. EMT and CSC models suggest that elimination of the EMT and/or CSC pool is required for effective inhibition of tumor metastasis and relapse. Therefore, targeting CSCs and EMT markers in combination with conventional cancer therapies will likely produce better long-term clinical outcomes (Figure 5). The dual EGFR/HER2 inhibitor LAPATINIB displayed potential in reducing the breast CSC population in neoadjuvant clinical trials [73]. Further, LAPATINIB in combination with conventional breast cancer therapies resulted in notable improvements in patient outcomes [73].

Conventional therapies target proliferating cells. The dose used to target cancer cells will also kill normal cells. Radio- and chemotherapy fails to kill CSCs. Therefore, regrowth of cancer is evident. CSC-targeted therapies may be much better than conventional therapies and may reduce the ability of cancer cells to generate. Combined treatments are more effective than individual therapies; targeting CSCs/EMT cells along with a low dose of conventional therapy was reported to be successful in most animal experiments. This approach will kill CSCs and resistant EMT cells along with differentiated and highly proliferating cancer cells, and helps prevent recurrence.

Transformed cancer cells that have undergone EMT exhibit CSC properties [48,49]. Therefore, targeting molecular activators of EMT, such the (PI3K)/AKT, Wnt/β-catenin, Notch, TGF-β, and NfKB pathways, and downstream transcriptional regulators of EMT, including Twist, ZEB1/2, and Snail family members, could prevent cancer cells from undergoing EMT and gaining an invasive phenotype. Therefore, inhibition of EMT or reversion of CSCs to a more differentiated epithelial phenotype by inducing MET may induce cell death or sensitize the cells to conventional therapies. Thus, the inhibition of EMT may help to inhibit tumor cell metastasis and the formation of CSCs. Furthermore, the signaling pathways regulating the process of EMT in various human cancers play roles in stem cell self-renewal and survival [56,57].

Agents targeting the pro-oncogenes and molecular signaling molecules associated with tumor progression and metastasis show promise in the treatment of cancer, but are inefficient, since patients inevitably develop resistance to these drugs [74]. However, recent advances in immunotherapies use the endogenous immune system to eliminate cancer cells and can produce long-term disease control and treatment-free survival [74,75]. Pre-clinical and early clinical trials have explored several potential strategies for immunotherapy for solid tumors. These strategies can be divided into two primary subcategories: 1) checkpoint inhibitors/immune modulators and 2) adoptive T cell transfer. Among these, NIVOLUMAB, a fully human immunoglobulin G4 programmed death 1 (PD-1) immune checkpoint inhibitor antibody, is the first to gain regulatory approval, and is now approved for use in patients [76]. Reports suggested that the molecular signaling pathways involved in regulating EMT and CSCs (NF-κB, MAPK, PI3K, mTOR, and JAK/STAT) upregulate PD-L1 in cancer cells [77,78]. However, many important issues surrounding the ideal duration of therapy and the role of retreatment with immunotherapeutic agents, particularly with NIVOLUMAB, are yet to be answered. Recent research strongly suggests combining immunotherapy agents with chemotherapy and molecular targeted therapy for effective treatment [76,79].

DCLK1 regulates the EMT process in pancreatic, liver, kidney, and colorectal cancers; knockdown of DCLK1 results in decreased cancer growth, EMT, and metastasis [9,11–15,66,80]. It is reported that DCLK1 regulates tumor cell self-renewal by controlling the expression of pluripotency factors [9,67]. Furthermore, the results of lineage tracing experiments showed that DCLK1 marks CSCs in pancreatic and colon cancer models [8,10,18]. Functional links between EMT, stemness, and DCLK1 are correlated with cancer resistance, metastasis, and recurrence. Together, these studies strongly suggest that DCLK1+ cells may be the cells of origin for cancer, and are suspected to be responsible for cancer recurrence and the development of deadly metastases that are resistant to standard therapies. Therefore, we suggest that (i) targeting DCLK1+ cells with a novel therapeutic approach using an innovative taxane antibody-drug conjugated (ADC) and (ii) inhibiting DCLK1 expression using novel DCLK1 inhibitors have the potential to reduce the process of cancer initiation, progression, and metastasis by regulating EMT and CSCs death, which will improve patients’ quality of life.

Together, these studies highlight the need to combine conventional therapies with drugs targeting the self-renewal, survival, and drug resistance of cells with an EMT/CSC phenotype. This approach may produce a more effective therapeutic strategy for improved cancer treatment. The genetic complexity of most human tumors indicates that knocking out a single target even in one specific cancer type is unlikely to produce sustained growth inhibition. New therapeutic strategies suggest that combination therapies are necessary for cancer growth control mechanisms that will result in successful treatment.

## Concluding Remarks

CSCs and EMT characteristics, which are critical components of treatment resistance, metastasis, and recurrence, are linked. The literature largely supports that EMT plays a critical role in supporting cancer stemness and/or CSCs, which leads to an increase in the metastatic potential and development of secondary cancer at distant sites. Therefore, a better understanding of the role of cancer self-renewal or CSCs and EMT phenotype in cancers will help determine more effective targeted therapies and/or combined therapies that will target not only the differentiated cancer cells, but also the subpopulation of cancer cells that are accountable for the metastasis, relapse, and reappearance of the cancer. Researchers have strongly suggested targeting DCLK1 for effective cancer treatment and improved survival. Therefore, the development of novel therapeutics that target DCLK1 may reduce CSCs and EMT, which may be a promising treatment strategy for the eradication of cancer without recurrence.
